# Distinctive pattern of AHNAK methylation level in peripheral blood mononuclear cells and the association with HBV‐related liver diseases

**DOI:** 10.1002/cam4.1778

**Published:** 2018-09-27

**Authors:** Libo Sun, Kang Li, Guihai Liu, Yuan Xu, Aiying Zhang, Dongdong Lin, Haitao Zhang, Xiaofei Zhao, Boxun Jin, Ning Li, Yonghong Zhang

**Affiliations:** ^1^ Department of Hepatobiliary Surgery Beijing You'an Hospital Capital Medical University Beijing China; ^2^ Biomedical Information Center Beijing You'an Hospital Capital Medical University Beijing China; ^3^ Department of Community Science University of Calgary Calgary Alberta Canada; ^4^ Beijing Institute of Hepatology Beijing You'an Hospital Capital Medical University Beijing China; ^5^ Beijing You'an Hospital Capital Medical University Beijing China

**Keywords:** AHNAK, liver diseases, methylation, methylation‐specific polymerase chain reaction, receiver operating characteristic curve

## Abstract

The purpose of this study was to investigate the correlation between AHNAK methylation level in peripheral blood mononuclear cells (PBMC) and the progression of hepatitis B virus (HBV)‐related liver disease. Bioinformatics methods were applied to evaluate the AHNAK methylation level in PBMC and T cells at different stages of HBV related liver disease, to investigate the correlation between AHNAK methylation and clinical features, as well as to compare the methylation site of AHNAK in cancer tissues and adjacent tissues. Subsequently, the differentially expressed gene analysis technique was used to analyze the liver disease‐related genes and immune‐related pathways in hepatitis B patients with different pathological changes. Finally, promoter methylation and mRNA expression of AHNAK gene in liver cancer and adjacent tissues were determined by quantitative polymerase chain reaction (Q‐PCR), and the diagnostic value of AHNAK methylation level in hepatopathy was evaluated by receiver operating characteristic (ROC) curve. The promoter methylation level of AHNAK gene in PBMCs decreased with the progression of HBV‐related liver disease, and showed significant difference among the patients with various HBV‐related liver diseases (*P *=* *0.0001). The AHNAK methylation level in PBMCs and T cells was negatively associated with age, white blood cell count, CREA, drinking, and positively associated with APTT and HbsAg. Higher mRNA expression of AHNAK was found in liver cancer tissues than that of adjacent tissues (*P *<* *0.001), and the methylation level in PBMC decreased with the progression of hepatitis B‐related liver disease. The area under the ROC curve (ROC) was 0.883 (*P *<* *0.001) in diagnosis of chronic hepatitis B (CHB), 0.885 (*P *<* *0.001) in diagnosis of compensatory liver cirrhosis, 0.955 (*P *<* *0.001) in diagnosis of decompensated liver cirrhosis, 0.981 (*P *<* *0.001) in diagnosis of hepatocellular carcinoma. Our results revealed that AHNAK methylation level in peripheral blood decreases with the progression of hepatitis B‐related liver disease. This provided a potential differential diagnostic method for HBV‐related hepatopathies, and thus an early detective tool for liver cancer.

## 1 INTRODUCTION

As one of the commonest malignant tumors, liver cancer ranks the fifth in morbidity and the third in mortality, with about 700 000 deaths worldwide every year.^1^, ^2^, ^3^ It is reported that liver cancer is associated with a dismal prognosis in most patients, and its five‐year survival rate is only about 10%.^4^ However, the five‐year survival rate of patients with early liver cancer can be up to 50%‐75%.^5^ Due to the lack of a singular effective clinical method for early diagnosis, patient with liver cancer is often diagnosed at an advanced stage and the best timing for curative treatment is missed. Therefore, it is of great significance to find effective biomarkers for early diagnosis and prognosis determination of liver cancer.^6^

Epigenetic modification is an important mechanism for the occurrence and development of many metabolic diseases and cancers.^7^, ^8^, ^9^, ^10^ DNA methylation is recognized as one of the first discovered epigenetic modifications, which can regulate gene expression by influencing chromatin structure, DNA conformation, DNA stability, and DNA‐protein action mode.^11^ A growing number of studies have shown that DNA methylation is associated with occurrence and prognosis of cancer and might be used as a diagnostic and prognostic molecular marker.^12^, ^13^ For instance, inhibition of HHIP promoter methylation could suppress human gastric cancer cell proliferation and migration^10^; GSTM2 methylation level can be served as a potential biomarker for breast cancer development^14^; hypermethylation of EphA5 promoter is an important marker for diagnosis and prognosis of pancreatic cancer.^15^ Our previous study found that the host immune system of liver cancer has unique DNA methylation characteristics, and significant changes of methylation in blood are expected to be noninvasive markers for early diagnosis of liver cancer.^16^ However, there are few studies on the abnormal DNA methylation of single gene in liver cancer.^17^

AHNAK is a desmosomal connexin, located in chromosome 11: 62433542‐62556235, encoding 700 kDa protein, and is expressed in all kinds of cancer cells.^18^, ^19^ It has been reported that low expression of AHNAK can effectively inhibit breast cancer cell invasion and proliferation as a tumor suppressor.^20^, ^21^ In glioma, AHNAK can depress tumor development and serve as a biomarker of tumor prognosis.^22^ Moreover, low expression of AHNAK in melanoma indicates poor prognosis.^23^ Nevertheless, the role of AHNAK in the development and prognosis of liver cancer remains unclear.

Therefore, this study utilizes bioinformatics to analyze the sequencing data from previous studies to identify the relationship of AHNAK methylation with the progression and immune‐related signaling pathways of HBV‐related liver diseases. The study results provided evidence for establishment of potential early diagnostic and prognostic biomarkers for liver cancer.

## 2 MATERIALS AND METHODS

### 2.1 Tissue(Blood) samples

In total, 242 Hepatitis B patients who were admitted into Beijing You'an Hospital, Capital Medical University during 1 June 2013 to 30 December 2016 were recruited. The study sample included 34 chronic hepatitis B (CHB), 32 compensatory liver cirrhosis (CLC), 35 decompensated liver cirrhosis (DCLC), 141 hepatocellular carcinoma (HCC) (stage 0 = 32; stage A = 38; stage B = 14; stage C = 36; stage D = 21) patients. As control, eighteen healthy volunteers were involved in this study. The method of obtaining PBMC and T cells refers to our previous study, which has been published.^16^ All participants and their family signed informed consents, and the study was approved by the medical research ethics committee of Beijing You'an Hospital, Capital Medical University (EC‐B‐031‐A02‐V9.0).

### 2.2 Data acquisition

Methylated pyrosequencing data were collected from previous studies.^16^ We obtained methylation data of hepatocellular carcinoma (HCC) from Cancer Genome Atlas (TCGA, https://gdc.cancer.gov/) platform. The gene expression dataset (GSE89377/E‐MTA‐950) was collected from the Gene Expression Omnibus (GEO, https://www.ncbi.nlm.nih.gov/geo/) database and array express (E‐MTAB‐950, https://www.ebi.ac.uk/arrayexpress) database. GEO dataset included 107 samples covering nine stages of HCC. We included 13 normal, 20 chronic Hepatitis (choronic), 12 cirrhosis (CS), five early hepatocellular carcinoma (eHCC) samples for analyses. E‐MTAB‐950 dataset included three chronic hepatitis with HBV (CHB) and 10 normal samples.

### 2.3 Gene expression and pathway analysis

The gene expression profile data were normalized by the Linear Models for Microarray Data (LIMMA, http://www.bioconductor.org/packages/release/bioc/html/limma.html) package in R. Also we used LIMMA to identify DEGs by comparing expression value between cancer and normal group samples. A Kyoto Encyclopedia of Genes and Genomes (KEGG) pathway 24 analysis was performed to evaluate the different expression of proteins on the regulation of TCR between different groups.

### 2.4 Network analysis

The Search Tool for the Retrieval of Interacting Genes (STRING), which provides information for experimental and predicted interactions, is an online database. STRING was applied to search and to determine an interaction network between AHNAK and TCR pathway.

### 2.5 DNA extraction, bisulfite modification, and methylation‐specific PCR (MSP)

**Figure 1:**
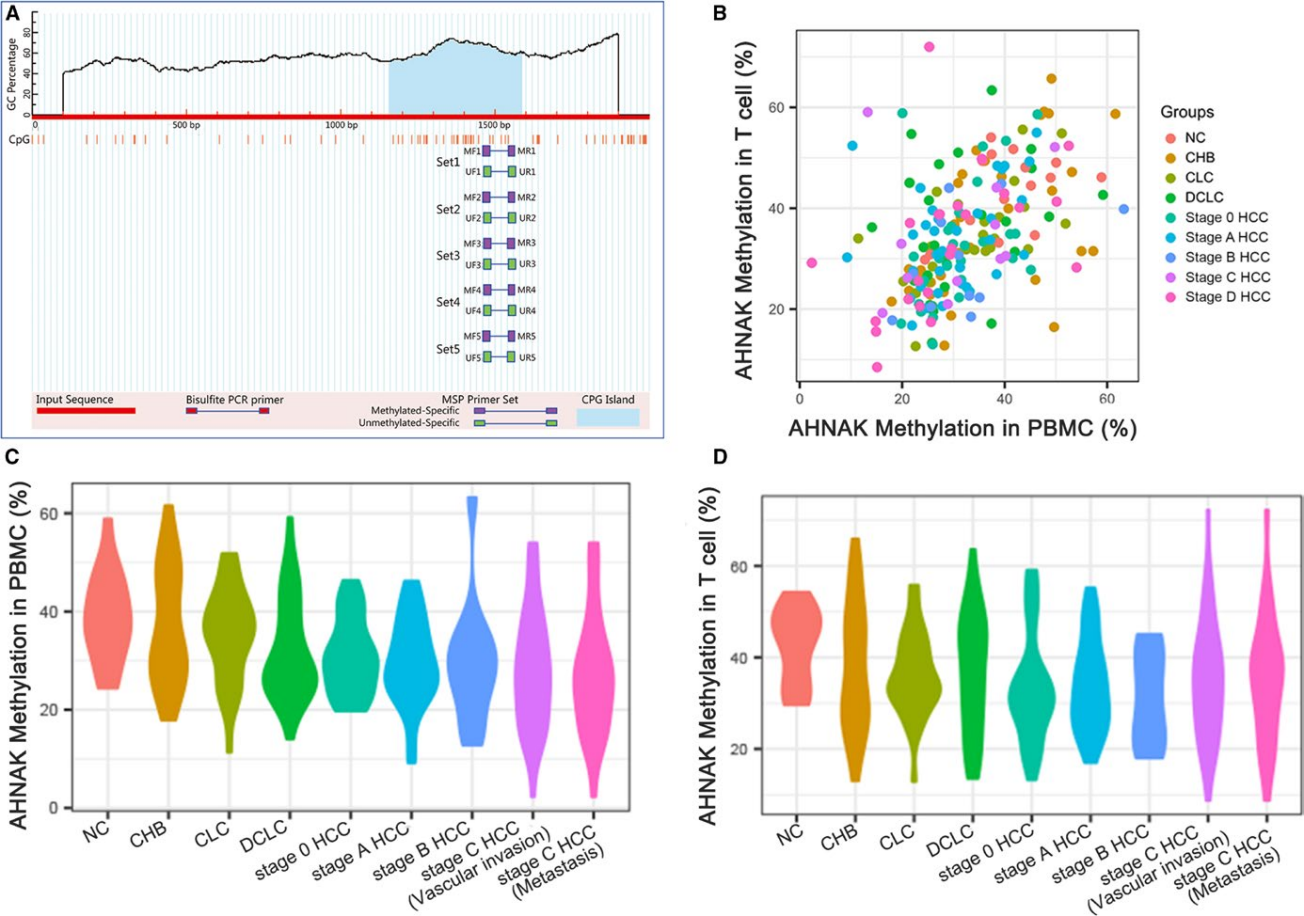
Methylation level of AHNAK promoter in PBMC and T cells in different liver diseases. A, Human AHNAK promoter was mapped to a base 337‐bp upstream of the gene, mainly containing one CpG island. B, distribution map of AHNAK promoter methylation level in PBMC and T cells at different disease stages; C, methylation level of AHNAK promoter in PBMC at different disease stages; D, methylation level of AHNAK promoter in T cells at different disease stages

Genomic DNA was extracted from peripheral blood mononuclear cells which derived from patients and controls using the QIAamp DNA mini kit (Qiagen NV, Venlo, Netherlands) in line with the manufacturer's protocol. The quality and quantity of the isolated DNA were measured by NanoDrop 2000 Spectrophotometer (Thermo Fisher Scientific). Bisulfite treatment of genomic DNA was processed using an EpiTect Fast DNA Bisulfite Kit (Qiagen NV). Bisulfite‐treated DNA samples were kept at −20°C for further use. After analyzing 2000‐bp region upstream of the transcription start site (AHNAK promoter region), one CpG island was identified (Figure 1A). With bisulfite‐treated DNA as the template, the methylation pattern in the CpG island within AHNAK promoter was measured by MSP, and MSP primers were designed as follows: [for methylated DNA F 5′‐ GTGGAAATTTAAGTTAGTTTTGTGC‐3′ and R 5′‐TTACTTAATTCCCAAACTCCGTA‐3′ and for unmethylated DNA F 5′‐ GGAAATTTAAGTTAGTTTTGTGTGG ‐3′ and R 5′‐CCTTACTTAATTCCCAAACTCCATA‐3′], according to the previously described principle.^25^ The reaction condition for MSP was 10 minutes at 95°C, followed by 40 cycles of 30 seconds at 94°C and 30 seconds at 72°C, and then 5‐min extension at 72°C. After separated on 1.5% agarose gels, the MSP products were then stained by ethidium bromide and visualized under UV spectrophotometer. Water blanks were served as negative control.

### 2.6 Sample preparation, RNA isolation, reverse transcription, and Q‐PCR

Total RNA was isolated using Trizol reagent (Invitrogen Carlsbad, CA, USA). cDNA was obtained from 2 μg total RNA by reverse transcription in accordance with the kit (Takara Biotechnology Ltd., Dalian, China). The cDNA was stored at −20°C. Real‐time quantitative PCR (RQ‐PCR) was performed using a GoTaq 2‐Step RT‐qPCR System (Promega, Madison, WI, USA) on ABI PRISM 7900 system (Applied Biosystems, Grand Island, NY, USA). The abundances of AHNAK mRNA were estimated by housekeeping gene β‐actin. Gene relative expression is analyzed by the 2^−∆∆Ct^ method.^26^ The experiments were performed in triplicate.

### 2.7 Statistical analysis

Student's *t* test and one‐way analysis of variance (ANOVA) were used to compare the level of AHNAK methylation between two and more groups, respectively. Spearman's correlation was used to assess the correlations between AHNAK methylation and continuous variables. Linear regression was utilized to evaluating the correlations between AHNAK methylation and discrete variables. Statistical significance was defined as a two‐tailed *P *<* *0.05 for all analyses. The ROC curve analysis was performed using SPSS19.0 (IBM Corporation, Chicago, IL, USA). Other analyses were conducted using R3.1.1 or GraphPad Prism version 6.0 (GraphPad software, San Diego California USA).

## 3 RESULTS

### 3.1 Promoter methylation level of AHNAK in PBMC was associated with the progression of HBV‐related liver disease

In order to understand the relationship between AHNAK methylation level and the progression of HBV related liver disease, we analyzed the methylation level in PBMC and T cells in different course samples. The results demonstrated that AHNAK methylation level showed no certain regularity in different samples (Figure 1B). In PBMC, the methylation level decreased as the severity of the disease increased, but this change was not obvious in T cells (Figure 1C,D). The statistical analysis suggested that AHNAK methylation level in PBMC showed significant difference among different groups (*F* value = 4.09, *P *<* *0.001), while no significant difference was found in T cells (*F* value = 0.197, *P *>* *0.05) (Table 1). There was significant difference in AHNAK methylation level in PBMC between control (NC) and DCLC groups, NC and HCC (stage 0, A, B, C, D) groups, CHB and HCC (stage 0, A, B, C, D) groups, CLC and HCC (stage 0, A, B, C, D) groups, respectively (Table 2).

**Table 1:** ANOVA analysis of methylation level of AHNAK gene in PBMC and T cells

	Df	Sum Sq	Mean Sq	*F* value	*P*r(>*F*)	Significant
PBMC	8	3609	451.2	4.090	0.0001	***
T cell	8	1605	200	1.398	0.197	ns

***p < 0.001.

**Table 2:** The methylation level of AHNAK gene in each group was analyzed by paired *t* test in PBMC

Group 1	Group 2	*P*	Significant
NC	CHB	0.5215	ns
NC	CLC	0.2177	ns
NC	DCLC	0.0207	*
NC	Stage 0	0.0035	**
NC	Stage A	0.0011	**
NC	Stage B	0.0240	*
NC	Stage C	0.0005	***
NC	Stage D	0.0005	***
CHB	CLC	0.5809	ns
CHB	DCLC	0.0864	ns
CHB	Stage 0	0.0177	*
CHB	Stage A	0.0056	**
CHB	Stage B	0.0638	ns
CHB	Stage C	0.0027	**
CHB	Stage D	0.0025	**
CLC	DCLC	0.1856	ns
CLC	Stage 0	0.0381	*
CLC	Stage A	0.0111	*
CLC	Stage B	0.1150	ns
CLC	Stage C	0.0055	**
CLC	Stage D	0.0053	**
DCLC	Stage 0	0.5430	ns
DCLC	Stage A	0.2656	ns
DCLC	Stage B	0.4495	ns
DCLC	Stage C	0.1250	ns
DCLC	Stage D	0.0863	ns
Stage 0	Stage A	0.5542	ns
Stage 0	Stage B	0.6743	ns
Stage 0	Stage C	0.2635	ns
Stage 0	Stage D	0.1754	ns
Stage A	Stage B	0.9225	ns
Stage A	Stage C	0.5441	ns
Stage A	Stage D	0.3606	ns
Stage B	Stage C	0.7795	ns
Stage B	Stage D	0.6010	ns
Stage C	Stage D	0.7229	ns

*p < 0.05, **p < 0.01, ***p < 0.001.

### 3.2 AHNAK methylation level in PBMC and T cells was pertinent to age, WBC count, CREA, APTT, drinking, and HBsAg

To further explore the correlation between AHNAK methylation level in PBMC and T cells and clinicopathological features of liver disease, Spearman's correlation and linear regression were applied. As shown in Tables S1 and S2, AHNAK methylation level in PBMC was correlated with age, WBC count, CREA, APTT, smoking, drinking, sex, and HBsAg; AHNAK methylation level in T cells was correlated with age, WBC count, CREA, APTT, drinking, and HBsAg.

### 3.3 Promoter methylation level of AHNAK was decreased in liver cancer tissues

To verify the reliability of the previous analysis, methylation data of hepatocellular carcinoma (HCC) collected from the cancer genome atlas (TCGA) database were processed by *t* test or Wilcoxon rank‐sum test. The findings showed that liver tissues displayed lower methylation level of AHNAK promoter than adjacent tissues (Tables S3 and S4).

### 3.4 Changes of related genes and signaling pathways in hepatitis B patients with different pathological changes

**Figure 2:**
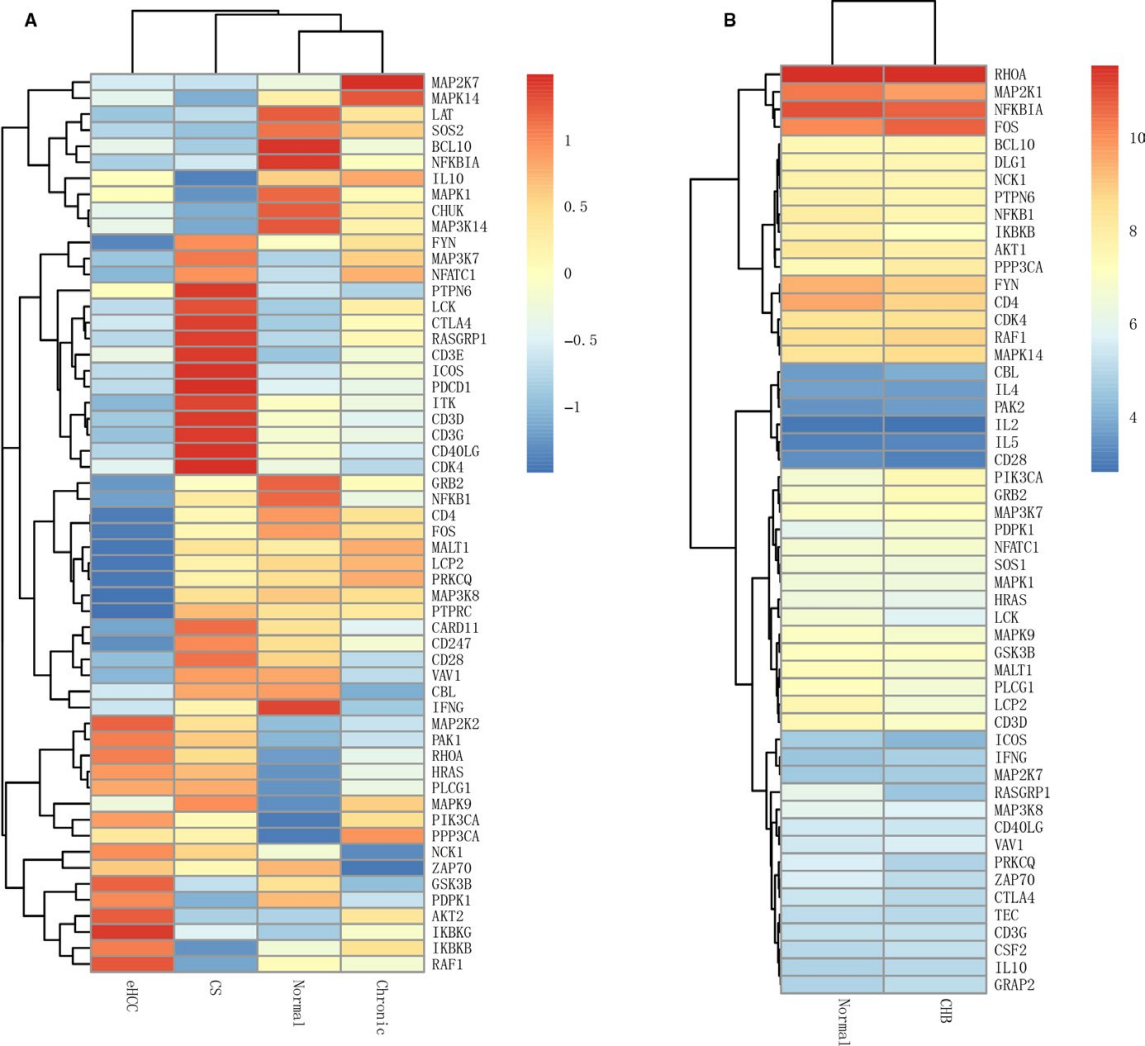
The heat map analysis for liver disease‐related genes. A, comparisons of liver disease‐related genes in eHCC, CS, Normal, and Chronic groups; B, comparisons of liver disease‐related genes in CHB and Normal groups. Note: Red indicates high expression, and green indicates low expression; the darker the color, the higher the expression. All the comparisons are compared to the Normal group

**Figure 3:**
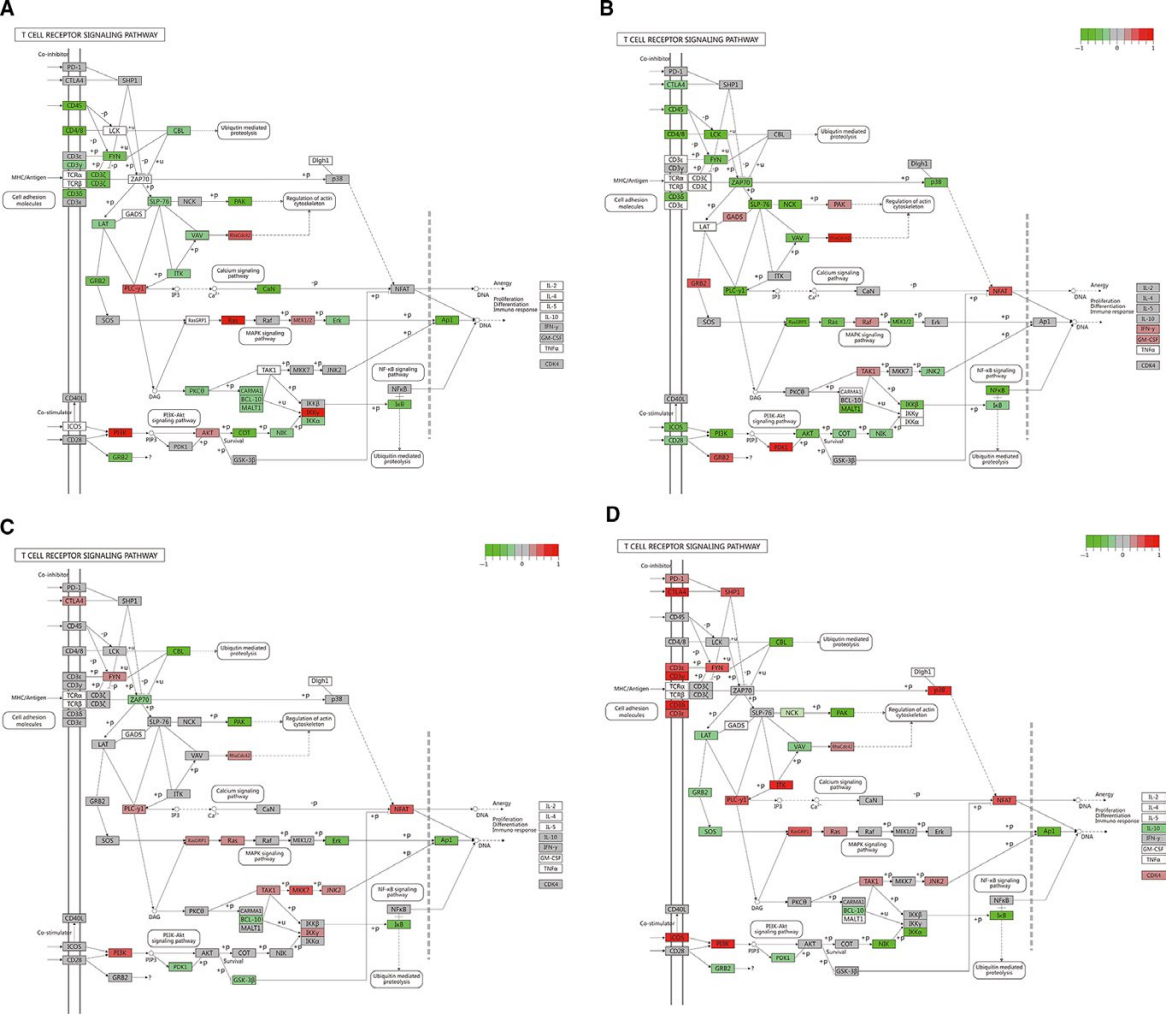
KEGG analysis for immune‐related pathway. A, Comparison of differentially expressed genes in T‐cell receptor signaling pathway between eHCC and normal groups. B, between CHB and normal groups. C, between chronic and normal groups. D, between CS and normal groups. Note: Red indicates up‐regulation, and green down‐regulation. KEGG, kyoto encyclopedia of genes and genomes

Subsequently, analysis for related genes and signaling pathways was carried out. As shown in Figure 2, MAP2K7 and MAPK14 were highly expressed in chronic hepatitis; LAT, SOS2, and BCL‐10 were highly expressed in normal tissues; PTPN6, LCK, and CTLA4 were highly expressed in CS. As shown in Figure 3, higher expressions of RHOA, MAP2K1, NFKBIA, and FOS were found in normal and HBV tissues. Compared with the normal group, there were higher expressions of Rho/Cdc42, PLC‐y1, Ras, and PI3K, while lower expressions of CD45, CD4/8, FγN, PAK, GRB2, and AP1 in the eHCC group (Figure 3A, Table 3); higher expressions of GRB2 and Rho/Cdc42, while lower expressions of CD45, CD4/8, MEK1/2, PI3K, p38, and AKT in the CHB group (Figure 3B, Table 3); higher expressions of Rho/Cdc42 and PI3K, while lower expressions of AP1, BCL‐10, and PDK1 in the Chronic group (Figure 3C, Table 3); higher expressions of CTLA4, SHP1, p38, and PI3K, while lower expressions of CBL, PAK, LAT, GRB2, and SOS in the CS group (Figure 3D, Table 3).

**Table 3:** Differential gene expression in different groups of immune‐related signaling pathways

Group	High expression	Low expression
eHCC vs normal	Rho/Cdc42; PLC‐y1; Ras; PI3K; IKKy	CD45; CD4/8; FγN; CD3ϭ; PAK; GRB2; AP1; COT; IкB
Choronic vs normal	CTLA4; FγN; Rho/Cdc42; NFAT; PI3K; MKK7	CBL; PAK; ZAP70; ErK; AP1; BCL‐10;PDK1;GSK‐3β; IкB
CS vs normal	CTLA4; SHP1; CD3δ/CD3ε/CD3γ/NCK; p38; ITK; ICOS; PI3K	CBL; PAK; LAT; GRB2; SOS; BCL‐10; AP1; IкB; NIK; IKKα
CHB vs normal	GRB2; Rho/Cdc42; NFAT; PDK1; Raf	CTLA4; CD45; CD4/8; LCK; FγN; CD3δ; SLP‐76;NCK;PLC‐γ1;Ras GRP1; Ras; p38; MEK1/2; PI3K; AKT; PKCB; MALT1; ICOS

### 3.5 AHNAK mRNA expression was elevated in liver cancer tissues and AHNAK methylation level in PBMC decreased with increasing severity of disease

**Figure 4:**
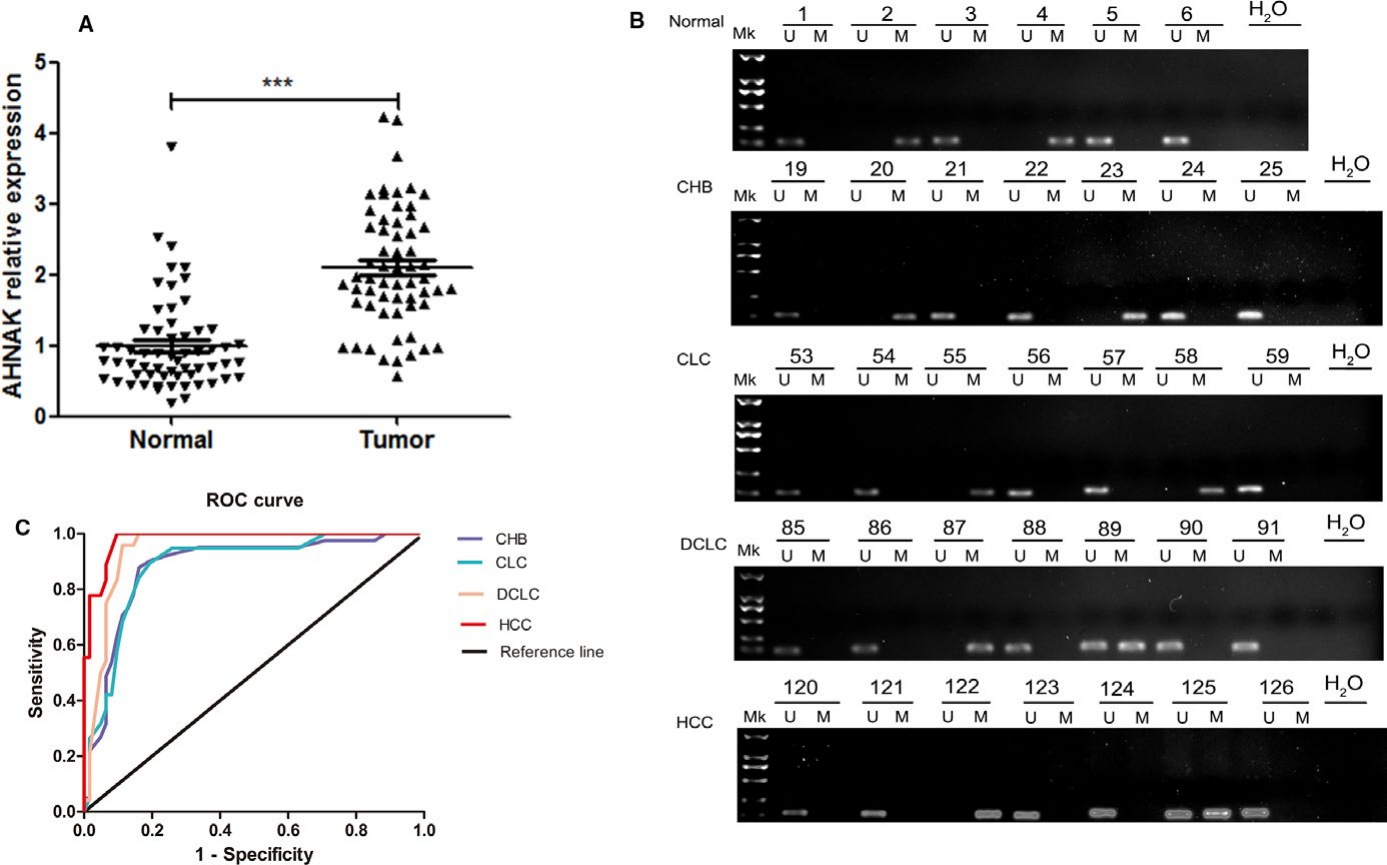
promoter methylation level of AHNAK in liver tissues and diagnostic value of AHNAK methylation. A, mRNA expression of AHNAK in liver tissues by Q‐PCR; values are expressed as mean ± SD (n = 60). *** indicates *P *<* *0.001. B, promoter methylation level of AHNAK in PBMC in patients with different liver diseases; C, Diagnostic value of AHNAK methylation level in liver disease. Note: Q‐PCR, quantitative polymerase chain reaction. M indicates methylation, and U demethylation. Mk, DNA marker. ROC, receiver operating characteristics

Next, an investigation of AHNAK mRNA expression was conducted in 60 cases of liver cancer tissues and adjacent tissues, and the methylation level of AHNAK promoter in PBMC was determined in 260 cases with different liver diseases. The results revealed that liver cancer tissues had significantly higher AHNAK mRNA expression than the adjacent tissues (*P* < 0.001, Figure 4A). There were 8 cases of methylation in NC group (44.44%), 13 cases in CHB group (38.23%), 11 in CLC group (34.38%), 11 cases in DCLC group (31.43%), 141 cases in HCC group (*P *<* *27.66%). It shows that the methylation level of AHNAK decreases with the increase of disease severity (Figure 4B, Table 4).

**Table 4:** Methylation level of AHNAK gene in liver disease

Group	Total number	Methylation	Percentage
NC	18	8	44.44
CHB	34	13	38.23
CLC	32	11	34.38
DCLC	35	11	31.43
HCC	141	141	27.66

### 3.6 Diagnostic value of AHNAK methylation level in patients with different liver diseases

To further verify whether AHNAK methylation in PBMC can be used as an indicator for the diagnosis of liver disease, the ROC curve was drawn for the analysis (Figure 4C). The results demonstrated that the AUC was 0.8828 in diagnosis of CHB, greater than the area below the reference line; the AUC was 0.8850 in diagnosis of CLC, greater than the area below the reference line (*P *<* *0.001); the AUC was 0.9459 in diagnosis of DCLC, greater than the area below the reference line (*P *<* *0.001); the AUC was 0.9807 in diagnosis of HCC, greater than the area below the reference line (*P *<* *0.001).

## 4 DISCUSSION

Liver cancer is one of the most frequent malignant neoplasia worldwide and is a multistage and multifactorial pathogenesis involving functional abnormalities of multiple genes.^27^, ^28^ Recently, epigenetic changes were found to have an influence on the development and prognosis of liver cancer.^29^, ^30^ It is well known that methylation of CpG island in the promoter region can silence stable genes by inhibiting the binding of transcription factors or recruiting methyl‐CpG binding proteins, which is conducive to the decrease or deletion of product expression of oncogene and further promoting apoptosis of cancer cells.^31^, ^32^ The level of DNA methylation is affected not only by DNA methyltransferases, but also by many factors, such as diet, environment, and living habits.^33^, ^34^ In this study, we found that age, WBC count, CREA, APTT, smoking, drinking, sex, and HBsAg could affect AHNAK methylation level in PBMC, and that age, WBC count, CREA, APTT, drinking, and HBsAg could affect AHNAK methylation level in T cells. Among them, drinking and smoking are the risk factors that induce the occurrence of primary liver cancer.^35^, ^36^ In addition, the results showed that AHNAK methylation level in PBMC decreases with the exacerbation of HBV related liver disease, suggesting that there is a certain correlation between AHNAK methylation and the progression of liver disease. However, the above trend was not obvious in T cells, which may be due to the limitation of sample size (Figure 1B,C). Although the diagnosis and treatment of liver cancer has been better developed, such as surgical resection and liver transplantation, the survival rate is still disappointing.^4^, ^31^ On cellular molecular level, both down‐regulated expression of mir‐33a and up‐regulated expression of TRIM14 suggest poor prognosis in patients with liver cancer.^37^, ^38^ In the current study, significantly decreased promoter methylation level and increased mRNA expression of AHNAK were shown in liver cancer tissues (all *P *<* *0.05), indicating that AHNAK methylation level can be served as a marker for the diagnosis of liver cancer.

Collectively, highly expression of AHNAK is associated with the occurrence of liver cancer, which is helpful to the early detection and diagnosis. However, the function and mechanism of AHNAK in liver cancer require further study.

## CONFLICTS OF INTEREST

The authors have declared that they have no conflict of interest.

## References

[1] Zhang F , Lee J , Liang S , Shum CK . Cyanobacteria blooms and non‐alcoholic liver disease: evidence from a county level ecological study in the United States. Environ Health. 2015;14:41. 25948281 10.1186/s12940-015-0026-7 PMC4428243

[2] Yang JD , Roberts LR . Hepatocellular carcinoma: a global view. Nat Rev Gastroenterol Hepatol. 2010;7(8):448‐458. 20628345 10.1038/nrgastro.2010.100 PMC3926946

[3] Wan MLY , El‐Nezami H . Targeting gut microbiota in hepatocellular carcinoma: probiotics as a novel therapy. Hepatobiliary Surg Nutr. 2018;7(1):11‐20. 29531939 10.21037/hbsn.2017.12.07 PMC5835615

[4] Qi J , Wang J , Katayama H , Sen S , Liu SM . Circulating microRNAs (cmiRNAs) as novel potential biomarkers for hepatocellular carcinoma. Neoplasma. 2013;60(2):135‐142. 23259781 10.4149/neo_2013_018 PMC3869230

[5] Vilana R , Forner A , Garcia A , Ayuso C , Bru C . Hepatocellular carcinoma: diagnosis, staging, and treatment strategy. Radiologia. 2010;52(5):385‐398. 20667565 10.1016/j.rx.2010.05.003

[6] Chen W , Zheng R , Baade PD , et al. Cancer statistics in China, 2015. CA Cancer J Clin. 2016;66(2):115‐132. 26808342 10.3322/caac.21338

[7] Karlsson IK , Ploner A , Wang Y , Gatz M , Pedersen NL , Hagg S . Apolipoprotein E DNA methylation and late‐life disease. Int J Epidemiol. 2018 10.1093/ije/dyy025. PMC7263750 29509901

[8] Mittelstrass K , Waldenberger M . DNA methylation in human lipid metabolism and related diseases. Curr Opin Lipidol. 2018;29(2):116‐124. 29517982 10.1097/MOL.0000000000000491 PMC5882251

[9] Verma M , Kumar V . Epigenetic biomarkers in colorectal cancer. Mol Diagn Ther. 2017;21(2):153‐165. 27878475 10.1007/s40291-016-0244-x

[10] Zuo Y , Lv Y , Qian X , et al. Inhibition of HHIP promoter methylation suppresses human gastric cancer cell proliferation and migration. Cell Physiol Biochem. 2018;45(5):1840‐1850. 29539620 10.1159/000487875

[11] Kumar S , Karmakar BC , Nagarajan D , Mukhopadhyay AK , Morgan RD , Rao DN . N4‐cytosine DNA methylation regulates transcription and pathogenesis in Helicobacter pylori. Nucleic Acids Res. 2018;46(7):3815. 29538771 10.1093/nar/gky195 PMC5909436

[12] Borssen M , Nordlund J , Haider Z , et al. DNA methylation holds prognostic information in relapsed precursor B‐cell acute lymphoblastic leukemia. Clin Epigenetics. 2018;10:31. 29515676 10.1186/s13148-018-0466-3 PMC5836434

[13] Fontecha‐Barriuso M , Martin‐Sanchez D , Ruiz‐Andres O , et al. Targeting epigenetic DNA and histone modifications to treat kidney disease. Nephrol Dial Transplant. 2018:1‐4. 29534238 10.1093/ndt/gfy009

[14] Kresovich JK , Gann PH , Erdal S , Chen HY , Argos M , Rauscher GH . Candidate gene DNA methylation associations with breast cancer characteristics and tumor progression. Epigenomics. 2018;10(4):367‐378. 29528252 10.2217/epi-2017-0119 PMC5925433

[15] Li S , Zhu Y , Ma C , et al. Downregulation of EphA5 by promoter methylation in human prostate cancer. BMC Cancer. 2015;15:18. 25609195 10.1186/s12885-015-1025-3 PMC4307617

[16] Zhang Y , Petropoulos S , Liu J , et al. The signature of liver cancer in immune cells DNA methylation. Clin Epigenetics. 2018;10:8. 29375724 10.1186/s13148-017-0436-1 PMC5774119

[17] Shen B , Jiang Y , Chen YR , et al. Expression and inhibitory role of TIMP‐3 in hepatocellular carcinoma. Oncol Rep. 2016;36(1):494‐502. 27222429 10.3892/or.2016.4818

[18] Davis TA , Loos B , Engelbrecht AM . AHNAK: the giant jack of all trades. Cell Signal. 2014;26(12):2683‐2693. 25172424 10.1016/j.cellsig.2014.08.017

[19] Shtivelman E , Cohen FE , Bishop JM . A human gene (AHNAK) encoding an unusually large protein with a 1.2‐microns polyionic rod structure. Proc Natl Acad Sci USA. 1992;89(12):5472‐5476. 1608957 10.1073/pnas.89.12.5472 PMC49314

[20] Lee IH , Sohn M , Lim HJ , et al. Ahnak functions as a tumor suppressor via modulation of TGFbeta/Smad signaling pathway. Oncogene. 2014;33(38):4675‐4684. 24662814 10.1038/onc.2014.69 PMC4180639

[21] Chen B , Wang J , Dai D , et al. AHNAK suppresses tumour proliferation and invasion by targeting multiple pathways in triple‐negative breast cancer. J Exp Clin Cancer Res. 2017;36(1):65. 28494797 10.1186/s13046-017-0522-4 PMC5427595

[22] Zhao Z , Xiao S , Yuan X , et al. AHNAK as a prognosis factor suppresses the tumor progression in glioma. J Cancer. 2017;8(15):2924‐2932. 28928883 10.7150/jca.20277 PMC5604443

[23] Sheppard HM , Feisst V , Chen J , Print C , Dunbar PR . AHNAK is downregulated in melanoma, predicts poor outcome, and may be required for the expression of functional cadherin‐1. Melanoma Res. 2016;26(2):108‐116. 26672724 10.1097/CMR.0000000000000228 PMC4777222

[24] da Huang W , Sherman BT , Lempicki RA . Systematic and integrative analysis of large gene lists using DAVID bioinformatics resources. Nat Protoc. 2009;4(1):44‐57. 19131956 10.1038/nprot.2008.211

[25] Li LC , Dahiya R . MethPrimer: designing primers for methylation PCRs. Bioinformatics. 2002;18(11):1427‐1431. 12424112 10.1093/bioinformatics/18.11.1427

[26] Livak KJ , Schmittgen TD . Analysis of relative gene expression data using real‐time quantitative PCR and the 2(‐Delta Delta C(T)) Method. Methods. 2001;25(4):402‐408. 11846609 10.1006/meth.2001.1262

[27] Zhang L , Chen J , Gao C , Liu C , Xu K . An efficient model for auxiliary diagnosis of hepatocellular carcinoma based on gene expression programming. Med Biol Eng Comput. 2018 10.1007/s11517-018-1811-6. [Epub ahead of print] 29546505

[28] Balogh J , Victor D 3rd , Asham EH , et al. Hepatocellular carcinoma: a review. J Hepatocell Carcinoma. 2016;3:41‐53. 27785449 10.2147/JHC.S61146 PMC5063561

[29] Bagnyukova TV , Tryndyak VP , Muskhelishvili L , Ross SA , Beland FA , Pogribny IP . Epigenetic downregulation of the suppressor of cytokine signaling 1 (Socs1) gene is associated with the STAT3 activation and development of hepatocellular carcinoma induced by methyl‐deficiency in rats. Cell Cycle. 2008;7(20):3202‐3210. 18843197 10.4161/cc.7.20.6816

[30] Thomson JP , Ottaviano R , Unterberger EB , et al. Loss of Tet1‐Associated 5‐Hydroxymethylcytosine Is Concomitant with Aberrant Promoter Hypermethylation in Liver Cancer. Can Res. 2016;76(10):3097‐3108. 10.1158/0008-5472.CAN-15-1910 PMC5021200 27197233

[31] Villanueva A , Hoshida Y , Battiston C , et al. Combining clinical, pathology, and gene expression data to predict recurrence of hepatocellular carcinoma. Gastroenterology. 2011;140(5):1501‐1512. e1502. 21320499 10.1053/j.gastro.2011.02.006 PMC3081971

[32] Maemura K , Yoshikawa H , Yokoyama K , et al. Delta‐like 3 is silenced by methylation and induces apoptosis in human hepatocellular carcinoma. Int J Oncol. 2013;42(3):817‐822. 23337976 10.3892/ijo.2013.1778 PMC3597457

[33] Donkin I , Barres R . Sperm epigenetics and influence of environmental factors. Mol Metab. 2018;14:1‐11. 10.1016/j.molmet.2018.02.006 PMC6034033 29525406

[34] Huang Y , Hui Q , Walker DI , et al. Untargeted metabolomics reveals multiple metabolites influencing smoking‐related DNA methylation. Epigenomics. 2018;10(4):379‐393. 29528243 10.2217/epi-2017-0101 PMC5925442

[35] Zhang YQ , Peng LJ , Cao YR , et al. Risk factors for hepatocellular carcinoma in cirrhotic patients with chronic hepatitis B. Genet Test Mol Biomarkers. 2016;20(9):535‐543. 27391584 10.1089/gtmb.2016.0062

[36] Costiniuk CT , Brunet L , Rollet‐Kurhajec KC , et al. Tobacco smoking is not associated with accelerated liver disease in human immunodeficiency virus‐hepatitis C coinfection: a longitudinal cohort analysis. Open Forum Infect Dis. 2016;3(2):ofw050. 27047987 10.1093/ofid/ofw050 PMC4817089

[37] Dong B , Zhang W . High levels of TRIM14 are associated with poor prognosis in hepatocellular carcinoma. Oncol Res Treat. 2018;41(3):129‐134. 29485416 10.1159/000485625

[38] Xie RT , Cong XL , Zhong XM , et al. MicroRNA‐33a downregulation is associated with tumorigenesis and poor prognosis in patients with hepatocellular carcinoma. Oncol Lett. 2018;15(4):4571‐4577. 29541227 10.3892/ol.2018.7892 PMC5835885

