# Supplementary material for: Distinctive pattern of AHNAK methylation level in peripheral blood mononuclear cells and the association with HBV‐related liver diseases
Source: Cancer Med. 2018 Sep 27;7(10):5178–86. doi: 10.1002/cam4.1778 (PMC6198198; doi:10.1002/cam4.1778)
Supplement: Supplementary file 1 [file CAM4-7-5178-s001.docx]

Table S1 Association analysis between AHNAK methylation and continuous clinical information in PBMC and T cells

| Feature | COR_PBMC | P_PBMC | significant | COR_T | P_T | significant |
| --- | --- | --- | --- | --- | --- | --- |
| Height | -0.0525 | 0.4577 | ns | -0.0585 | 0.3992 | ns |
| Weight | -0.0280 | 0.6925 | ns | -0.0327 | 0.6377 | ns |
| BMI | 0.0039 | 0.9568 | ns | -0.0070 | 0.9195 | ns |
| Age | -0.2364 | 0.0001 | *** | -0.2785 | 0.0000 | *** |
| HGB | -0.1061 | 0.0915 | ns | -0.1226 | 0.0475 | * |
| WBC | -0.1640 | 0.0088 | ** | -0.0593 | 0.3391 | ns |
| PLT | 0.03196 | 0.6122 | ns | 0.0171 | 0.7832 | ns |
| ALT | 0.09824 | 0.1183 | ns | -0.0041 | 0.9478 | ns |
| AST | 0.0937 | 0.1373 | ns | 0.0459 | 0.4603 | ns |
| TBIL | -0.0282 | 0.6546 | ns | 0.0236 | 0.7039 | ns |
| DBIL | -0.0160 | 0.8000 | ns | 0.0115 | 0.8526 | ns |
| TP | 0.0643 | 0.3073 | ns | 0.0296 | 0.6334 | ns |
| ALB | -0.0061 | 0.9232 | ns | 0.0387 | 0.5331 | ns |
| CREA | -0.1253 | 0.0465 | * | -0.0455 | 0.4650 | ns |
| CHOL | -0.0577 | 0.3787 | ns | -0.0076 | 0.9069 | ns |
| r_GT | -0.0545 | 0.4075 | ns | 0.0681 | 0.2935 | ns |
| ALP | 0.0178 | 0.7868 | ns | -0.0424 | 0.5128 | ns |
| PT | 0.0713 | 0.2904 | ns | 0.1517 | 0.0225 | * |
| PT_Percentage | -0.0635 | 0.3429 | ns | -0.1290 | 0.05129 | ns |
| INR | 0.0943 | 0.1585 | ns | 0.1528 | 0.0207 | * |
| APTT | 0.2268 | 0.0006 | *** | 0.0271 | 0.6829 | ns |
| Peritoneal_dropsy | -0.0501 | 0.4939 | ns | 0.0545 | 0.4515 | ns |
| Bilirubin | -0.0876 | 0.2308 | ns | -0.0692 | 0.3389 | ns |
| Albumin | 0.0485 | 0.5076 | ns | 0.0714 | 0.3235 | ns |
| PT_prolong | 0.1388 | 0.0569 | ns | 0.0793 | 0.2729 | ns |
| child_pugh | -0.0393 | 0.5909 | ns | 0.0030 | 0.9665 | ns |
| AFP | 0.0088 | 0.8940 | ns | 0.1521 | 0.0183 | ** |
| AFU | 0.0790 | 0.2309 | ns | 0.0587 | 0.3679 | ns |

Note: COR_PBMC indicates the Spearman correlation between the percentage of AHNAK methylation in PBMC and the clinical phenotype; P_PBMC indicates the pvauel value associated with PBMC; COR_T indicates the Spearman correlation between the percentage of AHNAK methylation in T cells and the clinical phenotype; P_T represents the correlation in the cells.

Table S2 Association analysis between AHNAK methylation and
discrete random variable clinical information in PBMC and T cells

| Feature | coefficient_PBMC | P_PBMC | significant | coefficient _T | P_T | significant |
| --- | --- | --- | --- | --- | --- | --- |
| Smoking  Previous vs. No | -3.1600 | 0.1900 | ns | 0.8600 | 0.7200 | ns |
| Smoking  Yes vs. No | -4.6900 | 0.0047 | ** | -1.7700 | 0.3100 | ns |
| Drinking  Occassion vs. No | -6.8700 | 0.2000 | ns | -7.5000 | 0.1400 | ns |
| Drinking  Previous vs. No | -2.3500 | 0.3100 | ns | 4.6900 | 0.03800 | * |
| Drinking  Yes vs. No | -5.7800 | 0.0100 | * | -5.9800 | 0.0110 | * |
| Sex Malevs. Female | -5.2600 | 0.0013 | ** | -0.0490 | 0.9800 | ns |
| HBSAg + vs. - | 6.9900 | 0.03100 | * | 9.1400 | 0.0260 | * |
| Anti_HBs + vs. - | -3.0800 | 0.2600 | ns | -3.3900 | 0.2900 | ns |
| Hbe_Ag + vs. - | 2.3300 | 0.3700 | ns | 0.6000 | 0.8400 | ns |
| Anti_Hbe + vs. - | -1.700 | 0.2700 | ns | -0.8800 | 0.6100 | ns |
| Anti_HBc + vs. - | -10.7700 | 0.3300 | ns | 0.2600 | 0.9800 | ns |

Note: coefficient_PBMC indicates that the percentage of AHNAK methylation in PBMC is relative to the linear regression regression of clinical phenotype; P_PBMC represents the pvauel value of the correlation in PBMC; coefficient_T indicates the relationship between AHNAK methylation in T cells and the clinical phenotype of linear regression.

Table S3 analysis of methylation differences between AHNAK gene and CpG locus in cancer and adjacent cancer by Student's t test

| CpG | Location | median_at | median_pt | median_pn | P_at | FDR_at | P_pt | FDR_pt |
| --- | --- | --- | --- | --- | --- | --- | --- | --- |
| cg10647704 | chr11:62508132-62508133 | 0.7813 | 0.8270 | 0.8490 | 0.0000 | 0.0000 | 0.0062 | 0.1621 |
| cg14789828 | chr11:62433696-62433697 | 0.5551 | 0.584 | 0.625 | 0.380 | 0.0000 | 0.0145 | 0.3473 |
| cg19337651 | chr11:62546511-62546512 | 0.0488 | 0.0509 | 0.0544 | 0.0167 | 0.1839 | 0.5914 | 1.0000 |
| cg18255584 | chr11:62459413-62459414 | 0.8619 | 0.8710 | 0.8763 | 0.4650 | 0.0001 | 0.0352 | 0.7385 |
| cg08491575 | chr11:62516950-62516951 | 0.3381 | 0.3236 | 0.3192 | 0.1061 | 0.6369 | 0.1552002 | 1.0000 |
| cg18341059 | chr11:62547035-62547036 | 0.0383 | 0.0360 | 0.0410 | 0.4403 | 1.0000 | 0.0535 | 1.0000 |
| cg19764555 | chr11:62547647-62547648 | 0.1494 | 0.1530 | 0.1814 | 0.0000 | 0.0005 | 0.2152 | 1.0000 |
| cg23447569 | chr11:62547848-62547849 | 0.2241 | 0.2240 | 0.3430 | 0.4420 | 0.0000 | 0.0000 | 0.0026 |
| cg06219806 | chr11:62541338-62541339 | 0.7043 | 0.7491 | 0.6985 | 0.8853 | 1.0000 | 0.0308 | 0.6772 |
| cg11127874 | chr11:62447524-62447525 | 0.9134 | 0.9270 | 0.9310 | 0.0000 | 0.0000 | 0.1161 | 1.0000 |
| cg06520521 | chr11:62555744-62555745 | 0.7885 | 0.8162 | 0.7943 | 0.0030 | 0.0420 | 0.7327 | 1.0000 |
| cg08160224 | chr11:62480987-62480988 | 0.8635 | 0.8711 | 0.8305 | 0.0681 | 0.4865 | 0.9033 | 1.0000 |
| cg27641076 | chr11:62444316-62444317 | 0.6162 | 0.6346 | 0.5641 | 0.0077 | 0.1000 | 0.0101 | 0.2535 |
| cg26699546 | chr11:62471778-62471779 | 0.9449 | 0.9487 | 0.9517 | 0.0288 | 0.2593 | 0.8760 | 1.0000 |
| cg03274456 | chr11:62505644-62505645 | 0.9159 | 0.9208 | 0.8691 | 0.3244 | 1.0000 | 0.1664 | 1.0000 |
| cg22625903 | chr11:62545811-62545812 | 0.0704 | 0.0754 | 0.0768 | 0.0007 | 0.0118 | 0.7954 | 1.0000 |
| cg19192377 | chr11:62441884-62441885 | 0.8939 | 0.9085 | 0.9105 | 0.0017 | 0.0287 | 0.4937 | 1.0000 |
| cg19902569 | chr11:62546457-62546458 | 0.0631 | 0.0618 | 0.0685 | 0.0017 | 0.0287 | 0.0014 | 0.0444 |
| cg08740862 | chr11:62546945-62546946 | 0.0194 | 0.0191 | 0.0187 | 0.0110 | 0.1323 | 0.3686 | 1.0000 |
| cg14171514 | chr11:62541020-62541021 | 0.5724 | 0.5920 | 0.3990 | 0.0000 | 0.0000 | 0.0000 | 0.0000 |
| cg20518446 | chr11:62547562-62547563 | 0.1141 | 0.0891 | 0.2119 | 0.0180 | 0.1839 | 0.1731 | 1.0000 |
| cg25272143 | chr11:62444518-62444519 | 0.6709 | 0.7110 | 0.5930 | 0.0000 | 0.0000 | 0.0004 | 0.0123 |
| cg16646354 | chr11:62543018-62543019 | 0.0276 | 0.0239 | 0.0219 | 0.0000 | 0.0014 | 0.1660 | 1.0000 |
| cg26055210 | chr11:62543280-62543281 | 0.0577 | 0.0514 | 0.0505 | 0.0000 | 0.0000 | 0.0224 | 0.5152 |
| cg16672189 | chr11:62516587-62516588 | 0.9386 | 0.9420 | 0.9510 | 0.0000 | 0.0000 | 0.0060 | 0.1621 |
| cg06861841 | chr11:62444642-62444643 | 0.5694 | 0.6060 | 0.4790 | 0.0000 | 0.0000 | 0.0000 | 0.0009 |
| cg26484813 | chr11:62544101-62544102 | 0.6677 | 0.7360 | 0.7540 | 0.0000 | 0.0000 | 0.0024 | 0.0685 |
| cg21038682 | chr11:62547348-62547349 | 0.0805 | 0.0850 | 0.1028 | 0.0608 | 0.4865 | 0.1875 | 1.0000 |
| cg12857638 | chr11:62444697-62444698 | 0.5052 | 0.5560 | 0.4290 | 0.0000 | 0.0000 | 0.0000 | 0.0000 |
| cg16509239 | chr11:62444959-62444960 | 0.4930 | 0.5131 | 0.5441 | 0.0021 | 0.0316 | 0.30698 | 1.0000 |
| cg09183450 | chr11:62541041-62541042 | 0.4340 | 0.4320 | 0.2920 | 0.0000 | 0.0000 | 0.0000 | 0.0027 |
| cg01910617 | chr11:62546005-62546006 | 0.0161 | 0.0143 | 0.0152 | 0.0005 | 0.0088 | 0.1608 | 1.0000 |
| cg00685014 | chr11:62444723-62444724 | 0.6060 | 0.6240 | 0.4970 | 0.0000 | 0.0000 | 0.0000 | 0.0000 |
| cg22134923 | chr11:62547402-62547403 | 0.1875 | 0.1878 | 0.2499 | 0.0001 | 0.0021 | 0.0499 | 0.1000 |
| cg22365167 | chr11:62539389-62539390 | 0.2150 | 0.2020 | 0.3440 | 0.0000 | 0.0000 | 0.0049 | 0.1361 |
| cg18825531 | chr11:62553664-62553665 | 0.2234 | 0.2110 | 0.1090 | 0.0000 | 0.0000 | 0.0000 | 0.0016 |
| cg11489918 | chr11:62545248-62545249 | 0.0404 | 0.0437 | 0.0451 | 0.1555 | 0.7775 | 0.3483 | 1.0000 |
| cg05427381 | chr11:62542235-62542236 | 0.7401 | 0.7602 | 0.6693 | 0.4045 | 1.0000 | 0.2986 | 1.0000 |

Note: median_at means the median of this locus in at, similar to median_pt and median_pn. P_at and FDR_at means the P value and FDR for the at and PN groups of the t test (or Wilcoxon rank sum test). Group: at: all tumor; PT: tumor with normal control; PN: normal control.

Table S4 Analysis of methylation differences between AHNAK gene and CpG locus in cancer and adjacent cancer by Wilcoxonranksum

| CpG | Location | median_at | median_pt | median_pn | P_at | FDR_at | P_pt | FDR_pt |
| --- | --- | --- | --- | --- | --- | --- | --- | --- |
| cg10647704 | chr11:62508132-62508133 | 0.7813 | 0.8270 | 0.8490 | 0.0000 | 0.0000 | 0.0118 | 0.2401 |
| cg14789828 | chr11:62433696-62433697 | 0.5551 | 0.5840 | 0.6250 | 0.0021 | 0.0406 | 0.0500 | 0.9007 |
| cg19337651 | chr11:62546511-62546512 | 0.0488 | 0.0509 | 0.0544 | 0.0073 | 0.1016 | 0.3344 | 1.0000 |
| cg18255584 | chr11:62459413-62459414 | 0.8619 | 0.8710 | 0.8763 | 0.0125 | 0.1123 | 0.0585 | 0.9943 |
| cg08491575 | chr11:62516950-62516951 | 0.3381 | 0.3236 | 0.3192 | 0.6593 | 1.0000 | 0.3062 | 1.0000 |
| cg18341059 | chr11:62547035-62547036 | 0.0383 | 0.0360 | 0.0410 | 0.0596 | 0.3576 | 0.0114 | 0.2401 |
| cg19764555 | chr11:62547647-62547648 | 0.1494 | 0.1530 | 0.1814 | 0.0000 | 0.0003 | 0.0079 | 0.1826 |
| cg23447569 | chr11:62547848-62547849 | 0.2241 | 0.2240 | 0.3430 | 0.0000 | 0.0000 | 0.0000 | 0.0024 |
| cg06219806 | chr11:62541338-62541339 | 0.7043 | 0.7491 | 0.6985 | 0.6505 | 1.0000 | 0.0043 | 0.1111 |
| cg11127874 | chr11:62447524-62447525 | 0.9134 | 0.9270 | 0.9310 | 0.0000 | 0.0001 | 0.5116 | 1.0000 |
| cg06520521 | chr11:62555744-62555745 | 0.7885 | 0.8162 | 0.7943 | 0.8918 | 1.0000 | 0.9231 | 1.0000 |
| cg08160224 | chr11:62480987-62480988 | 0.8635 | 0.8711 | 0.8305 | 0.0646 | 0.3577 | 0.5888 | 1.0000 |
| cg27641076 | chr11:62444316-62444317 | 0.6162 | 0.6346 | 0.5641 | 0.0084 | 0.1016 | 0.0094 | 0.2071 |
| cg26699546 | chr11:62471778-62471779 | 0.9449 | 0.9487 | 0.9517 | 0.0096 | 0.1027 | 0.9923 | 1.0000 |
| cg03274456 | chr11:62505644-62505645 | 0.9159 | 0.9208 | 0.8691 | 0.0000 | 0.0000 | 0.0009 | 0.0285 |
| cg22625903 | chr11:62545811-62545812 | 0.0704 | 0.075 | 0.0768 | 0.0025 | 0.0444 | 0.6570 | 1.0000 |
| cg19192377 | chr11:62441884-62441885 | 0.894 | 0.9085 | 0.9105 | 0.0027 | 0.0460 | 0.9461 | 1.0000 |
| cg19902569 | chr11:62546457-62546458 | 0.0632 | 0.0618 | 0.0685 | 0.0006 | 0.0121 | 0.0033 | 0.0935 |
| cg08740862 | chr11:62546945-62546946 | 0.0194 | 0.0191 | 0.0187 | 0.1917 | 0.7668 | 0.8773 | 1.0000 |
| cg14171514 | chr11:62541020-62541021 | 0.5724 | 0.5920 | 0.3990 | 0.0000 | 0.0000 | 0.0000 | 0.0003 |
| cg20518446 | chr11:62547562-62547563 | 0.1141 | 0.0891 | 0.2119 | 0.0000 | 0.0001 | 0.0061 | 0.1467 |
| cg25272143 | chr11:62444518-62444519 | 0.6709 | 0.7110 | 0.5930 | 0.0000 | 0.0016 | 0.0006 | 0.0196 |
| cg16646354 | chr11:62543018-62543019 | 0.0276 | 0.0239 | 0.0219 | 0.0093 | 0.1027 | 0.1156 | 1.0000 |
| cg26055210 | chr11:62543280-62543281 | 0.0576 | 0.0514 | 0.0505 | 0.0000 | 0.0001 | 0.0611 | 0.9943 |
| cg16672189 | chr11:62516587-62516588 | 0.9386 | 0.9420 | 0.9510 | 0.0000 | 0.0000 | 0.0012 | 0.0354 |
| cg06861841 | chr11:62444642-62444643 | 0.5694 | 0.6060 | 0.4790 | 0.0003 | 0.0057 | 0.0001 | 0.0033 |
| cg26484813 | chr11:62544101-62544102 | 0.6677 | 0.7360 | 0.7540 | 0.0053 | 0.0797 | 0.0162 | 0.3084 |
| cg21038682 | chr11:62547348-62547349 | 0.0805 | 0.0850 | 0.1028 | 0.0008 | 0.0163 | 0.1156 | 1.0000 |
| cg12857638 | chr11:62444697-62444698 | 0.5052 | 0.5560 | 0.4290 | 0.0000 | 0.0004 | 0.0000 | 0.0002 |
| cg16509239 | chr11:62444959-62444960 | 0.4930 | 0.5131 | 0.5441 | 0.0288 | 0.2305 | 0.2313 | 1.0000 |
| cg09183450 | chr11:62541041-62541042 | 0.4340 | 0.4320 | 0.2920 | 0.0000 | 0.0000 | 0.0002 | 0.0084 |
| cg01910617 | chr11:62546005-62546006 | 0.016 | 0.0143 | 0.0152 | 0.0044 | 0.0700 | 0.1616 | 1.0000 |
| cg00685014 | chr11:62444723-62444724 | 0.6060 | 0.6240 | 0.4970 | 0.0000 | 0.0001 | 0.0000 | 0.0005 |
| cg22134923 | chr11:62547402-62547403 | 0.1875 | 0.1878 | 0.2499 | 0.0000 | 0.0003 | 0.0038 | 0.1021 |
| cg22365167 | chr11:62539389-62539390 | 0.2151 | 0.2020 | 0.3440 | 0.0000 | 0.0000 | 0.0045 | 0.1135 |
| cg18825531 | chr11:62553664-62553665 | 0.2234 | 0.2110 | 0.1090 | 0.0000 | 0.0022 | 0.0005 | 0.0163 |
| cg11489918 | chr11:62545248-62545249 | 0.0404 | 0.0437 | 0.0451 | 0.0073 | 0.1016 | 0.9615 | 1.0000 |
| cg05427381 | chr11:62542235-62542236 | 0.7401 | 0.7602 | 0.6693 | 0.0373 | 0.2611 | 0.0968 | 1.0000 |

Table S5 AUC of different disease

| disease | AUC |
| --- | --- |
| CHB | 0.8828 |
| CLC | 0.8850 |
| DCLC | 0.9459 |
| HCC | 0.9807 |
